# Writing is thinking: toward a neuropsychological model of scientific writing as knowledge construction

**DOI:** 10.3389/fcogn.2026.1932224

**Published:** 2026-09-23

**Authors:** Jorge Alexander Ríos-Flórez, Albeiro de Jesús Muñoz-Giraldo, Alexander Rodríguez Bustamante, Tatiana Castañeda-Quirama

**Affiliations:** 1 Faculty of Law and Forensic Sciences, Tecnológico de Antioquia University Institution, Medellín, Colombia; 2 Faculty of Law and Political Science, Luis Amigó Catholic University, Medellín, Colombia; 3 School of Psychology, Institución Universitaria Politécnico Grancolombiano, Medellín, Colombia

**Keywords:** executive functions, knowledge construction, metacognition, neuropsychology, neuroscience, scientific writing

## Abstract

Scientific writing has historically been conceptualized as a communicative act—the registration of knowledge elaborated through independent cognitive processes. This article challenges that assumption and proposes that scientific writing constitutes an active neurocognitive system of knowledge construction and a privileged domain for metacognitive self-regulation in advanced learning contexts. We present the Integrated Neuropsychological Model of Scientific Writing (MNIEC), a theoretical framework integrating four neurocognitive systems—the Constructive Executive System (CES), the Extended Working Memory System (EWMS), the Semantic Simulation System (SSS), and the Epistemological Metacognitive Monitoring System (EMMS)—within a unified functional architecture. The central mechanism is the Epistemological Construction Cycle (ECC), a recursive five-phase process—conceptual activation, working memory simulation, symbolic externalization, metacognitive evaluation, and conceptual reorganization—through which scientific writing can function as an active mechanism of knowledge construction, particularly when recursive metacognitive evaluation and conceptual reorganization are engaged. The model integrates evidence from executive function neuropsychology, working memory architecture, large-scale brain network dynamics, epistemological metacognition, and predictive coding, grounded in five nuclear principles. Four testable hypotheses and experimental paradigms are derived. Implications are discussed for metacognitive training in scientific education, academic impostor syndrome, and the cognitive impact of large language models. The MNIEC proposes a neuropsychological framework for a systematic research agenda on scientific writing as metacognitive knowledge construction.

## 1 Introduction

For centuries, scientific writing has been conceptualized primarily as a communicative act—a mechanism through which researchers report findings produced via prior, independent cognitive processes. Under this view, the written text constitutes the terminal output of thought rather than a constitutive component of it. However, converging evidence from cognitive neuropsychology, functional neuroimaging, and the cognitive science of writing increasingly challenges this assumption. A substantial body of research suggests that writing does not merely express knowledge: it actively constructs it (Kellogg, 2008; Klein, 1999; Olive, 2014). This constructive function, however, is not uniform across all writing activities: it is most pronounced when writing involves recursive metacognitive monitoring, conceptual reorganization, and the integration of new representational structures—conditions that characterize high-level scientific writing rather than routine reporting or reformatting of stabilized ideas. This reconceptualization carries profound implications for the understanding of scientific cognition and, by extension, for researcher training and the evaluation of scientific productivity.

From a neurobiological standpoint, scientific writing recruits a highly demanding functional architecture involving the coordinated activation of multiple large-scale neural systems. The coordinated activation of the dorsolateral prefrontal cortex (DLPFC), the frontoparietal control network, perisylvian language areas, and medial prefrontal regions associated with metacognitive monitoring positions scientific writing as an emergent property of high-order neural integration, rather than a simple transcription process (Miller and Cohen, 2001; Menon and Uddin, 2010; Fleming and Dolan, 2012). Understanding these mechanisms is both theoretically relevant and a necessary condition for developing evidence-based interventions in scientific education and for critically evaluating the cognitive impact of automated text-generation technologies. Moreover, scientific writing constitutes one of the most demanding metacognitive learning tasks researchers engage in throughout their careers—a process in which the regulation of one's own epistemic processes is not incidental but constitutive of the knowledge being produced.

The contemporary relevance of this question has been underscored by a recent editorial in *Nature Reviews Bioengineering*, which argues that writing constitutes an irreplaceable component of the scientific method—not merely as a vehicle for communication, but as the cognitive process through which scientists think, organize evidence, and generate new knowledge (Editorial, 2025). Outsourcing writing to large language models (LLMs) may deprive researchers of this generative cognitive function, regardless of the surface quality of the text produced. This observation calls for a neuropsychological account of why writing is thinking—and not merely a metaphor for it.

Despite the richness of the existing literature on executive functions (Miyake et al., 2000), working memory (Baddeley, 2000, 2012), large-scale brain networks (Menon and Uddin, 2010), and metacognition (Fleming and Dolan, 2012), no integrative neuropsychological model has been proposed to date that formally articulates these systems within a unified architecture specifically applied to scientific writing.

The present paper addresses this gap by proposing the Integrated Neuropsychological Model of Scientific Writing (MNIEC, from its Spanish acronym)—a theoretical framework that conceptualizes scientific writing as an emergent neurocognitive system arising from the recursive interaction among four functional subsystems: the Constructive Executive System (CES), the Extended Working Memory System (EWMS), the Semantic Simulation System (SSS), and the Epistemological Metacognitive Monitoring System (EMMS). The model further proposes that these systems operate through iterative cycles of conceptual activation, symbolic externalization, and metacognitive evaluation—a process we term the Epistemological Construction Cycle (ECC)—enabling the progressive reorganization of cognitive representations and, under conditions of sustained metacognitive engagement, the emergence of new scientific knowledge that would not have been accessible without the writing process itself. In doing so, the MNIEC positions scientific writing as a privileged domain for the study and training of metacognitive self-regulation in advanced learning contexts.

The paper is organized as follows. Section 2 reviews the theoretical foundations underlying each of the four MNIEC subsystems, identifying their neurobiological substrates and specific contributions to scientific writing. Section 3 presents the formal architecture of the model, including its nuclear principles, functional phases, and proposed neurobiological bases. Section 4 derives four testable hypotheses from the model and proposes experimental paradigms for empirical validation. Section 5 discusses the theoretical implications of the MNIEC, its relationship to existing models, and its relevance in the context of AI-assisted writing. Concluding remarks are presented in Section 6.

## 2 Theoretical background

### 2.1 Executive functions and scientific writing

Executive functions (EFs) constitute a set of high-level cognitive processes that enable the flexible regulation of goal-directed behavior. The factorial model proposed by Miyake et al. (2000) identified three core components: updating of working memory representations, inhibition of prepotent responses, and shifting between mental sets. Although relatively independent, these three components share a common executive substrate and significantly predict performance across complex cognitive tasks.

Scientific writing represents one of the most demanding manifestations of executive functioning, requiring the deliberate planning of argumentative structures, the hierarchical organization of conceptual content, the inhibition of irrelevant information, and the continuous monitoring of the logical coherence of the text produced (Olive, 2014). Unlike everyday writing, scientific writing places simultaneous demands on all three executive components identified by Miyake et al. (2000): the writer must continuously update the mental model of the text, inhibit non-pertinent conceptual associations, and flexibly shift between global and local levels of discourse representation.

From a neurobiological standpoint, evidence directly establishes that executive functioning critically depends on the dorsolateral prefrontal cortex (DLPFC), a region that sustains the active maintenance and manipulation of online representations, as well as the selection of contextually relevant information (Miller and Cohen, 2001). The DLPFC operates in coordination with the anterior cingulate cortex (ACC), which participates in cognitive conflict detection and in the dynamic adjustment of executive control (Botvinick et al., 2004). These findings are well established in the executive function literature. By reasonable extrapolation to the scientific writing domain, the MNIEC proposes that this frontocingulate coordination constitutes the neural core of constructive executive control during writing—a claim that requires direct verification through paradigms specifically designed for scientific writing contexts.

Empirical evidence reinforces this link between executive functions and writing quality. Using structural equation modeling with secondary school students, Limpo and Alves (2013) demonstrated that planning and self-regulation skills—processes closely tied to the central executive—significantly predicted text generation quality beyond the contribution of transcription skills, and that this relationship strengthened with the writer's developmental level. Converging evidence from Graham and Harris (2000) documented that executive self-regulation is a core component of writing development, whose absence systematically compromises the conceptual quality of the text produced, irrespective of the writer's general linguistic competence. Neuroimaging research has documented activations within the frontoparietal network—including the DLPFC, premotor cortex, and inferior parietal lobule—during planning and production of written texts (Purcell et al., 2011). It should be noted that this evidence concerns typed spelling and reading rather than scientific discourse production specifically; its relevance to the MNIEC is therefore that of a suggestive extrapolation rather than direct support.

Taken together, the available evidence positions executive functions not as auxiliary processes but as a necessary condition for knowledge construction through writing—the central regulatory system of scientific writing.

### 2.2 Working memory and extended cognition

Working memory (WM) is the cognitive system responsible for the active maintenance and online manipulation of information during the execution of complex tasks. The multicomponent model proposed by Baddeley (2000, 2012) describes WM as an architecture comprising four subsystems: the central executive, which regulates attention and coordinates the slave subsystems; the phonological loop, which processes and maintains verbal information; the visuospatial sketchpad, which operates on visual and spatial representations; and the episodic buffer, which integrates information from multiple sources into coherent multimodal representations linked to long-term memory.

Scientific writing places particularly high demands on WM, requiring the simultaneous maintenance of multiple conceptual representations—the macrostructure of the argument, local coherence between sentences, rhetorical goals, and epistemological validity criteria—while continuously producing text (Kellogg, 2008). Kellogg demonstrated that expert writers distribute WM resources more efficiently across planning, translation, and revision processes, resulting in higher conceptual quality of the text produced, whereas novice writers saturate WM with low-level demands—spelling, syntax, punctuation—thereby compromising the capacity available for higher-order conceptual processing.

A theoretically pivotal contribution comes from Clark and Chalmers (1998), who proposed the extended mind principle: cognitive resources are not confined within the skull but are distributed across the nervous system and functionally integrated external artifacts. Under this principle, writing is not simply a product of thought but a functional extension of WM: by externalizing representations in symbolic form, the writer reduces internal cognitive load and enables the processing of conceptual structures of greater complexity than would be possible through purely internal processes.

WM thus does not merely support scientific writing as a passive temporary storage resource—it functions as an active platform for conceptual construction whose functional capacity is amplified through the symbolic externalization that writing makes possible. Empirical evidence supports this account: the externalization of working memory processes into external tools boosts immediate task performance, though the cognitive consequences depend critically on whether the writer actively engages in forming new conceptual representations during the offloading process (Grinschgl et al., 2021). Applied to scientific writing, this finding suggests that the knowledge-constructive function of externalization—formalized in the MNIEC as the EWMS's symbolic amplification mechanism—depends on the concurrent engagement of metacognitive monitoring, not on externalization alone. This principle constitutes one of the central pillars of the model proposed here.

### 2.3 Neural networks of scientific writing

Scientific writing does not depend on an isolated brain region but on the coordinated activation of a distributed neural network integrating executive control, semantic processing, linguistic production, and metacognitive monitoring.

Functional magnetic resonance imaging (fMRI) studies have identified activation of the dorsolateral prefrontal cortex (DLPFC) during written production tasks, including typed spelling and reading (Purcell et al., 2011)—evidence that, while not specific to scientific discourse production, suggests a role for DLPFC in the executive control of written language processes more broadly. This region operates in coordination with the left inferior frontal gyrus—Broca's area (BA44/BA45)—a structure critical for lexical selection, syntactic integration, and the formulation of conceptually articulated propositions (Bookheimer, 2002), and with the left superior temporal gyrus—Wernicke's area—which sustains semantic retrieval and meaning integration during text generation (Binder et al., 2009). The angular gyrus (BA39), located at the temporoparietal junction, constitutes a convergence node among the linguistic, semantic, and executive systems, mediating the multimodal integration of semantic representations during complex text production (Seghier, 2013).

From a large-scale functional network perspective, evidence establishes that cognition in general involves the dynamic interaction among three principal networks (Menon and Uddin, 2010): the frontoparietal control network (FPCN), responsible for maintaining cognitive goals and selecting relevant information; the default mode network (DMN), implicated in semantic retrieval from long-term memory, the simulation of conceptual scenarios, and the construction of an internal narrative that integrates memory, language, and conceptual representations (Menon, 2023); and the salience network (SN), whose central node—the anterior cingulate cortex—regulates the alternation between the FPCN and DMN. Importantly, the DMN is not a functionally homogeneous system: distinct subsystems make complementary contributions to semantic cognition, with medial temporal subsystems supporting retrieval of context-dependent knowledge and frontotemporal subsystems supporting controlled semantic access—a differentiation that has direct implications for the MNIEC's characterization of the SSS and its interaction with the CES (Shao et al., 2024). The MNIEC specifically predicts that these network dynamics operate during scientific writing in the manner described below, though this prediction has not yet been directly tested in scientific writing populations.

During idea generation phases, evidence from mind-wandering and creative cognition research suggests that the DMN retrieves prior knowledge and simulates conceptual structures; during task-focused processing, the FPCN suppresses the DMN and assumes executive control (Smallwood et al., 2012). The SN arbitrates this alternation by detecting cognitive conflict and shifting attentional resources accordingly. The MNIEC predicts that these dynamics apply to the specific phases of the ECC during scientific writing—a prediction that constitutes Hypothesis 1 of the model (see Figure 1). Direct electrophysiological evidence has confirmed the causal role of DMN dynamics in internally directed cognition, demonstrating that DMN engagement is actively regulated rather than merely correlational—a finding that supports the MNIEC's prediction that DMN suppression during ECC Phases 3 and 4 reflects active executive gating by the SN rather than passive disengagement (Bartoli et al., 2024).

**Figure 1:**
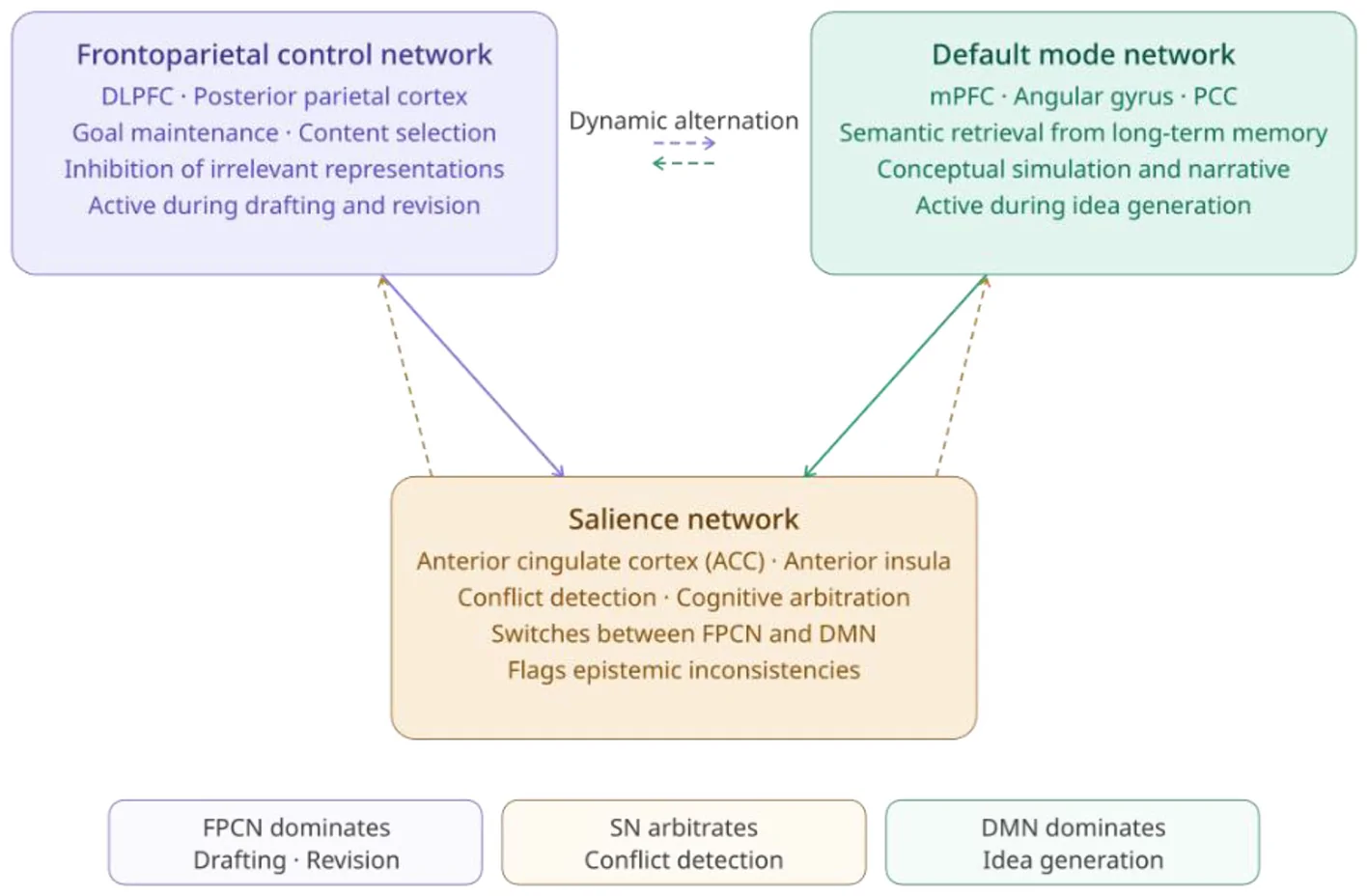
Dynamic interactions among large-scale brain networks during scientific writing. The frontoparietal control network (FPCN) dominates during drafting and revision; the default mode network (DMN) supports semantic retrieval and conceptual simulation during idea generation. The salience network (SN) arbitrates dynamic alternation between FPCN and DMN by detecting cognitive conflict and epistemic inconsistencies. Dashed arrows indicate network suppression/switching signals. DLPFC, dorsolateral prefrontal cortex; mPFC, medial prefrontal cortex; PCC, posterior cingulate cortex; ACC, anterior cingulate cortex. Figure of own elaboration based on Menon and Uddin (2010) and Smallwood et al. (2012). This figure is a theoretical schematic representing the MNIEC's proposed network dynamics during scientific writing. It does not depict empirically validated neural circuits specific to scientific writing; the depicted relationships constitute model-derived predictions subject to empirical verification.

Additional evidence comes from high-density electroencephalography research. Van der Weel and Van der Meer (2024) demonstrated that handwriting, compared with keyboard writing, produces greater distributed brain connectivity, with more extensive activations across temporal, parietal, and frontal regions, suggesting that the modality of externalization influences the neural architecture of the underlying cognitive process.

Recent neuroimaging research has confirmed that creative writing performance is predicted by resting-state functional connectivity patterns involving the default mode and executive control networks, with semantic memory structure—specifically, the breadth and interconnectedness of semantic categories accessible to the writer—acting as a mediating factor between neural connectivity and writing quality (Gu et al., 2025). This finding is directly consistent with the MNIEC's proposed interaction between the SSS and the FPCN during Phases 1 and 2 of the ECC. Scientific writing thus recruits, in a coordinated and dynamic manner, a distributed set of neural systems integrating executive control, semantic processing, linguistic production, and cognitive monitoring—a functional architecture whose specific organization during scientific discourse production remains to be directly characterized.

### 2.4 Epistemological metacognition

Metacognition—defined as the capacity to monitor, evaluate, and regulate one's own cognitive processes—constitutes a fundamental component of advanced scientific cognition. Flavell (1979) distinguished between metacognitive knowledge—what an individual knows about their own mental processes—and metacognitive regulation—the capacity to supervise and adjust those processes in real time. This distinction is particularly relevant in scientific writing, where the writer must continuously evaluate the validity, internal coherence, and epistemological adequacy of the knowledge being generated.

From a neurobiological standpoint, evidence directly establishes that metacognition depends on a medial prefrontal system comprising the medial prefrontal cortex (mPFC) and the rostral anterior cingulate cortex (rACC), regions implicated in introspective monitoring and in the evaluation of epistemic confidence regarding one's own representations (Fleming and Dolan, 2012). A recent comprehensive review and synthesis of metacognitive neuroscience confirms that the anterior prefrontal cortex and its connections with domain-specific processing regions constitute the core neural substrate of confidence-based epistemic self-assessment across diverse cognitive domains—a foundation that directly informs the neurobiological specification of the EMMS in the MNIEC (Fleming, 2024). Fleming et al. (2010) demonstrated that metacognitive efficiency correlates positively with gray matter volume in the anterior prefrontal cortex (BA10) and with the density of connections between this region and the inferior frontal gyrus—findings established in general metacognitive tasks. By extrapolation, the MNIEC proposes that these same substrates underlie epistemological monitoring during scientific writing, with individual variability in metacognitive efficiency predicting variability in writing quality—a claim formalized as Hypothesis 3.

In the context of writing, metacognition operates across multiple simultaneous levels: at the local level, the writer monitors coherence between adjacent propositions; at the global level, consistency between the argumentative macrostructure and the epistemological goals of the text is evaluated (Hacker et al., 2009). A central contribution to the MNIEC is the distinction drawn by Fleming and Dolan (2012) between first-order metacognition—monitoring the accuracy of specific representations—and second-order metacognition—evaluating the conceptual framework itself, including its assumptions, biases, and limits. Scientific writing requires both levels: the scientist must assess not only whether a claim is correct, but whether the conceptual framework from which it is formulated is epistemologically adequate. This process corresponds to what the MNIEC will term second-order epistemological monitoring.

Additional empirical evidence comes from research on reflective writing. Rivard (1994) documented that scientific writing—unlike other modes of knowledge recording—actively promotes metacognitive thinking by compelling the individual to render explicit representations that would otherwise remain implicit in internal thought. Converging with this view, Klein (1999) identified the facilitation of metacognitive monitoring as one of the central mechanisms through which writing generates new knowledge, beyond merely communicating pre-existing knowledge.

Writing externalizes thought and transforms it into an observable cognitive object—an artifact that the author can examine with critical distance. This process, which the MNIEC will formalize as symbolic externalization with metacognitive function, transforms writing into an environment of active epistemological evaluation: the written text is not merely the product of thought but the mirror in which thought observes and reorganizes itself.

### 2.5 Toward an integrated neuropsychological model

The four sections reviewed above converge on a common conclusion: scientific writing is an active neurocognitive system of knowledge construction, not a process of transcribing previously elaborated ideas. Executive functions organize and regulate the process; working memory—extended through externalization—provides the platform for conceptual manipulation; distributed brain networks sustain the integration of these systems; and metacognition evaluates and reorganizes the knowledge generated.

Despite the strength of this domain-specific evidence, the literature lacks an integrative neuropsychological model that articulates these four systems within a unified functional architecture, specifies their dynamic interactions during scientific writing, and generates testable hypotheses regarding their neurobiological bases. Existing writing models—including Hayes and Flower (1980), Kellogg (2008), and the parallel-cascading model of Olive (2014)—address partial dimensions of the process without offering a comprehensive neurocognitive integration. The Integrated Neuropsychological Model of Scientific Writing is proposed to address this theoretical gap (see Figure 2). An important epistemological clarification is warranted at this point. The MNIEC is a hypothesis-generating theoretical model, not an empirically validated account of the neurocognitive architecture of scientific writing. Its neurobiological specifications are derived from convergent but largely indirect evidence—principally from studies on general writing, executive functions, working memory, metacognition, and large-scale brain networks in non-scientific writing contexts. No study to date has directly investigated the integrated neural architecture of scientific writing as conceptualized here. Accordingly, throughout this paper, a systematic distinction is maintained among three levels of evidential status: (a) empirically established findings from the source literatures; (b) reasonable extrapolations from adjacent domains that motivate the model's architecture; and (c) predictions specific to the MNIEC that constitute its empirically testable claims. This distinction is intended to prevent the theoretical architecture of the model from being read as a description of already-demonstrated mechanisms.

**Figure 2:**
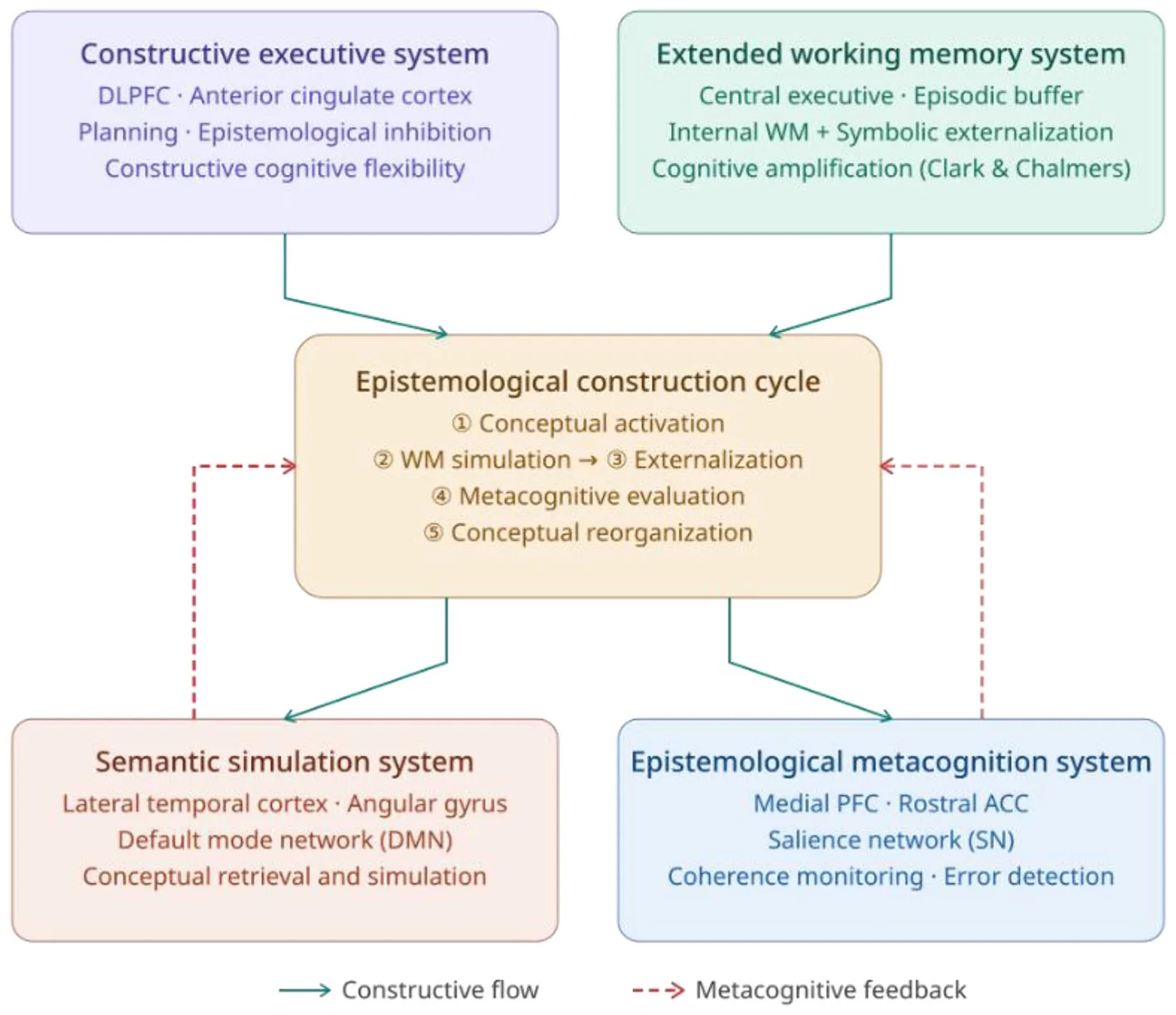
Theoretical framework of the MNIEC: neurocognitive systems and their dynamic interactions. The diagram represents the four main neurocognitive systems of the Integrated Neuropsychological Model of Scientific Writing (MNIEC) and their interaction through the Epistemological Construction Cycle (ECC). Solid arrows indicate constructive flow between systems; dashed arrows indicate metacognitive feedback. DLPFC, dorsolateral prefrontal cortex; WM, working memory; PFC, prefrontal cortex; ACC, anterior cingulate cortex; DMN, default mode network; SN, salience network. Figure of own elaboration based on Baddeley (2000), Miller and Cohen (2001), Fleming and Dolan (2012), and Menon and Uddin (2010). This figure is a theoretical schematic of the MNIEC's proposed architecture. The depicted systems, connections, and neurobiological substrates represent model-derived constructs and predictions, not empirically validated mechanisms of scientific writing.

## 3 The integrated neuropsychological model of scientific writing (MNIEC)

### 3.1 Nuclear principles of the model

The MNIEC is grounded in five nuclear principles that delineate its epistemological assumptions and guide its functional architecture:

1. *Principle 1***—***Epistemic emergence*: scientific knowledge does not pre-exist writing as a finished representation; it emerges progressively and non-linearly through the dynamic interaction among the model's four systems. Writing is a condition of possibility for knowledge, not its subsequent registration.
2. *Principle 2***—***Constructive recursivity*: the process of scientific writing is not sequential but recursive. Conceptual activation generates externalization; externalization triggers metacognitive evaluation; metacognitive evaluation reorganizes representations and restarts the cycle. This recursivity is not a flaw in the process—it is its central generative mechanism.
3. *Principle 3***—***Symbolic amplification*: the externalization of thought in written form is not a simple discharge of internal cognitive content. Following the extended mind principle (Clark and Chalmers, 1998), the written text functions as a functional extension of the cognitive system, amplifying the capacity for conceptual manipulation beyond the limits of internal working memory.
4. *Principle 4***—***Affective-motivational modulation*: the executive, metacognitive, and semantic processes constituting the MNIEC do not operate in a purely rational space. The amygdala, the orbitofrontal cortex, and the dopaminergic reward system actively modulate the availability and efficiency of the cognitive resources engaged in scientific writing (Pessoa, 2008). Writing anxiety, epistemic motivation, and tolerance for ambiguity are not peripheral contextual variables—they are first-order neurobiological determinants of system performance.
5. *Principle 5***—***Expertise sensitivity*: the MNIEC operates qualitatively differently depending on the writer's developmental level. In novice writers, the saturation of working memory with low-level demands (spelling, syntax, formal conventions) compromises the capacity available for higher-order conceptual processing. In expert writers, the automatization of low-level processes frees executive and metacognitive resources for epistemological construction of greater complexity (Kellogg, 2008).

These five principles collectively define the epistemological commitments of the MNIEC. It bears emphasis that the model operates at the level of theoretical specification: the principles identify the structural conditions under which scientific writing functions as knowledge construction, but their neurobiological instantiation remains to be directly verified through the experimental paradigms outlined in Section 4.

### 3.2 Functional architecture of the MNIEC: the four systems

The MNIEC proposes that scientific writing emerges from the coordinated interaction of four functionally specialized and neurobiologically differentiated neurocognitive systems. Each system makes a specific contribution to the overall process, yet none is sufficient on its own to account for the complexity of expert scientific writing.

1. *System 1***—***Constructive Executive System (CES)*. The CES integrates the core executive functions—updating, inhibition, and shifting (Miyake et al., 2000)—into an active regulatory process oriented toward the construction of scientific discourse. Its primary neurobiological substrate is the DLPFC, which maintains active the representations relevant to the epistemological goals of the text, suppresses non-pertinent conceptual associations, and implements flexible shifting between global and local levels of discourse representation (Miller and Cohen, 2001). The anterior cingulate cortex acts as a cognitive conflict detector, signaling discrepancies between generated representations and the writer's rhetorical and epistemological goals (Botvinick et al., 2004). The CES regulates not only what is written, but how it is argumentatively constructed. Empirically, CES engagement during scientific writing may be indexed by behavioral markers such as pre-writing planning time, structured outlining behavior, and keystroke-logging patterns showing extended pauses before major argumentative transitions—indicators of active goal maintenance and inhibitory control. Neurobiologically, CES activity is predicted to correlate with DLPFC and ACC activation during phases of deliberate argumentative construction, as specified in Hypothesis 1.
2. *System 2***—***Extended Working Memory System (EWMS)*. The EWMS articulates the multicomponent working memory architecture (Baddeley, 2000, 2012) with the extended cognition principle (Clark and Chalmers, 1998). Internally, the central executive coordinates the phonological loop—which maintains the linguistic representation of the text under construction—and the episodic buffer—which integrates semantic information from long-term memory with active textual representations. Externally, the written text functions as an extended working memory: by externalizing conceptual representations in symbolic form, the writer reduces internal cognitive load and enables the manipulation of knowledge structures of greater complexity than would be accessible through purely internal processes. This cognitive amplification constitutes one of the central neuropsychological mechanisms through which writing generates new knowledge. Empirically, EWMS function during writing may be indexed by the degree to which writers re-read previously produced text before continuing—a behavior detectable through eye-tracking and keystroke logging—and by the argumentative complexity of text produced under conditions of varying externalization availability, as operationalized in Hypothesis 2. Individual differences in WM capacity, measured through reading span tasks, are predicted to moderate the degree of reliance on external text as a cognitive scaffold.
3. *System 3***—***Semantic Simulation System (SSS)*. The SSS supports the retrieval of prior knowledge from long-term semantic memory and its active simulation within the cognitive workspace. Its neurobiological substrate includes the left lateral temporal cortex, the angular gyrus (BA39), and the default mode network (DMN), which activates during spontaneous idea generation, the retrieval of conceptual narratives, and the simulation of alternative epistemic scenarios (Binder et al., 2009; Smallwood et al., 2012). The SSS does not retrieve information passively: it actively simulates possible conceptual relations, generates implicit hypotheses, and feeds the ECC with semantic material that the CES and EWMS process, evaluate, and transform into articulated knowledge. Empirically, SSS engagement may be indexed by the semantic richness and conceptual diversity of idea-generation output prior to drafting, by the frequency of spontaneous conceptual associations reported in think-aloud protocols, and by DMN activation during unconstrained pre-writing ideation phases, as predicted in Hypothesis 1. Deficits in SSS function—corresponding to Type II Failure—may manifest behaviorally as extended pre-writing latencies with minimal productive output, low lexical diversity in early drafts, and self-reported semantic blocking.
4. System 4**—***Epistemological Metacognitive Monitoring System (EMMS)*. The EMMS constitutes the second-order evaluation and regulation system of the MNIEC. Its central function is the continuous monitoring of the internal coherence of the text produced, the evaluation of the epistemological validity of the claims generated, and the detection of inconsistencies between the writer's mental model and the externalized representations. Its neurobiological substrate includes the medial prefrontal cortex (mPFC) and the rostral anterior cingulate cortex (rACC)—associated with introspective processing and the evaluation of epistemic confidence (Fleming and Dolan, 2012)—and the anterior insula, the central node of the salience network, which regulates the alternation between generative processing (SSS/DMN) and evaluative processing (CES/FPCN) (Menon and Uddin, 2010). These are established findings in their respective source literatures, here extrapolated to the scientific writing context. Following the distinction drawn by Fleming and Dolan (2012), the EMMS operates at two levels: first-order metacognition evaluates the accuracy of specific representations, while second-order metacognition evaluates the adequacy of the conceptual framework from which the text is constructed—a process we term second-order epistemological monitoring, constituting a differential capacity of the expert scientific writer. Empirically, EMMS engagement during scientific writing may be indexed by revision density—the proportion of text that is revised relative to total text produced—by the structural depth of revisions (surface corrections vs. conceptual reorganizations), by pausing patterns at section boundaries detectable through keystroke logging, and by metacognitive confidence ratings collected during writing. At the neurobiological level, EMMS activity is predicted to correlate with mPFC and rACC activation during revision and evaluation phases, as specified in Hypothesis 1. Individual differences in metacognitive efficiency, measured through the meta-d‘/d' ratio, are predicted to account for significant variance in EMMS behavioral markers and in overall writing quality, as formalized in Hypothesis 3.

### 3.3 The epistemological construction cycle (ECC)

The four systems of the MNIEC operate neither in independent parallel nor in linear sequence. Their interaction generates a recursive and overlapping set of five functional phases that constitutes the central operational mechanism of the model: the Epistemological Construction Cycle (ECC). These phases are presented sequentially for descriptive clarity, but their actual operation during scientific writing is neither strictly linear nor discretely bounded—phases may co-occur, overlap, and operate simultaneously at different temporal scales, from the level of a single sentence to the level of the complete manuscript.

Within the predictive coding framework (Friston, 2010), the ECC can be understood as an iterative process of epistemological prediction error minimization operating across at least three representational levels. At the local level, the system generates predictions about propositional coherence—whether the next sentence or argument will be consistent with the conceptual representations currently active in working memory. At the discourse level, predictions concern the global argumentative structure—whether the developing text will satisfy the rhetorical and epistemological goals that motivated the writing episode. At the epistemic level, predictions concern the validity and justifiability of the conceptual framework itself—whether the model of the domain that the writer is developing is adequate to the evidence and reasoning being assembled. Externalization (Phase 3) constitutes the test of these predictions: the written text, once produced, generates prediction errors at each level when discrepancies arise between what was anticipated and what was actually expressed. These errors drive Phase 4 (metacognitive evaluation) and, when sufficiently resolved, Phase 5 (conceptual reorganization). This cycle does not terminate in the correction of surface errors—it terminates in the progressive reduction of epistemic uncertainty across all three representational levels. This account is further supported by theoretical work on cross-representational dynamics, which proposes that high-level cognition characteristically emerges through the coordination of symbolic, situational, and verbal representational formats—each imposing distinct constraints on the information being processed, and together generating cognitive elaboration not achievable within any single representational mode (Khatin-Zadeh et al., 2025). This principle is not specific to any single domain: it reflects a general feature of advanced knowledge construction through representational transformation. Applied to the MNIEC, it illuminates why Phase 3 (symbolic externalization) is epistemologically productive: translating internal conceptual representations into written symbolic form is not a neutral transcription but a representational transformation that imposes linguistic, logical, and rhetorical constraints—and it is precisely these constraints that generate prediction errors at the discourse and epistemic levels that drive Phase 5 (conceptual reorganization).

The five phases of the ECC are as follows:

1. *Phase 1***—***Conceptual activation*: the SSS retrieves relevant semantic representations from long-term memory and makes them available to the system. The CES selects and hierarchically organizes the representations pertinent to the epistemological goals of the text, inhibiting irrelevant material.
2. *Phase 2***—***Working memory simulation*: the EWMS integrates the activated representations within the episodic buffer, articulates them with prior knowledge, and simulates possible argumentative structures. In this phase, the writer mentally rehearses conceptual configurations before externalizing them.
3. *Phase 3***—***Symbolic externalization*: the simulated representations are translated into written text through the coordinated action of the phonological loop, the motor system, and the language areas (Broca's area, Wernicke's area, premotor cortex). The externalized text is not a copy of the internal representation—it is a transformation that imposes linguistic, rhetorical, and logical constraints on the conceptual content.
4. *Phase 4***—***Metacognitive evaluation*: the EMMS evaluates the text produced at two levels: local coherence (between adjacent propositions) and global coherence (between the argumentative macrostructure and the epistemological goals). The salience network detects discrepancies and activates the CES to initiate revision. This phase is neurobiologically costly—requiring the coordinated activation of the mPFC, rACC, and FPCN—which accounts for the cognitive fatigue characteristic of intensive scientific writing sessions.
5. Phase 5**—***Conceptual reorganization*: the inconsistencies detected in Phase 4 do not produce merely superficial textual corrections. In the expert writer, they trigger a reorganization of conceptual representations in long-term memory—the revised text reflects a genuinely modified mental model. This is the phase in which writing can generate new knowledge: not through the mechanical production of text, but through the cognitive reorganization that sustained metacognitive evaluation produces. This generative function is conditional—it is activated when the writer engages in genuine epistemic evaluation rather than surface-level editing, and it varies as a function of writing expertise, task demands, and metacognitive engagement.

Rather than restarting as a discrete sequence upon completion of Phase 5, the ECC operates as a continuously active, nested process: at any given moment during scientific writing, multiple instances of the cycle may be running concurrently at different scales—at the level of the sentence, the paragraph, the section, and the complete manuscript—with phases overlapping, interrupting, and recursively influencing one another. The sequential presentation of the five phases in this paper reflects an analytical decomposition for theoretical and empirical tractability, not a claim about the temporal architecture of actual writing behavior (see Figure 3).

**Figure 3:**
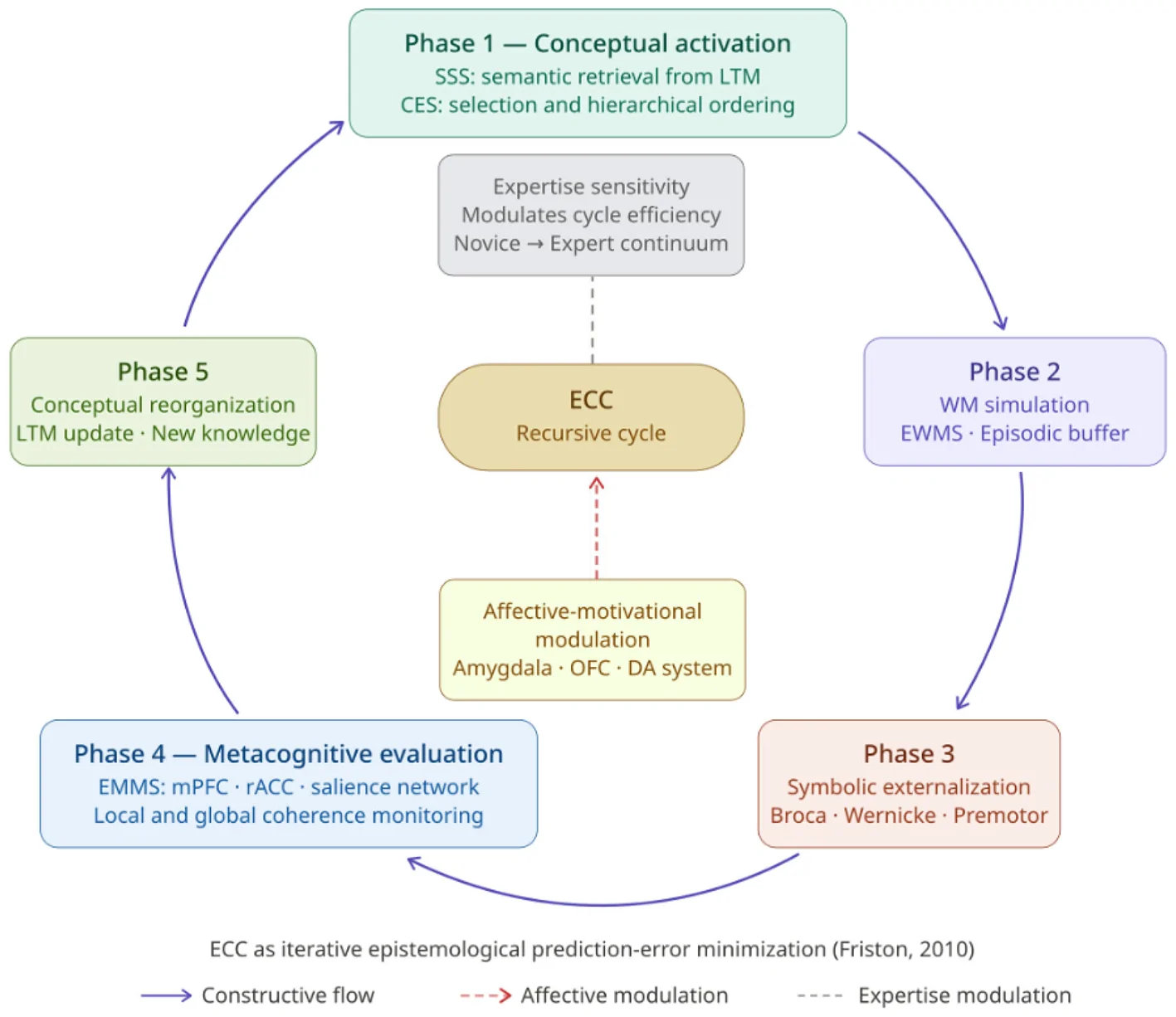
The Epistemological Construction Cycle (ECC): five functional phases and their neurocognitive systems. The ECC operates as a continuously active, nested, and overlapping process across four simultaneous scales: sentence, paragraph, section, and full manuscript. Solid arrows indicate the direction of the recursive cycle. The dashed arrow indicates transversal affective-motivational modulation exerted by the amygdala, orbitofrontal cortex (OFC), and dopaminergic reward system on all five phases. The expertise continuum modulates overall cycle efficiency. Interpreted within the predictive coding framework (Friston, 2010), the ECC constitutes a process of iterative minimization of epistemological prediction error. SSS, Semantic Simulation System; CES, Constructive Executive System; EWMS, Extended Working Memory System; EMMS, Epistemological Metacognitive Monitoring System; mPFC, medial prefrontal cortex; rACC, rostral anterior cingulate cortex; LTM, long-term memory; OFC, orbitofrontal cortex; DA, dopaminergic. This figure is a theoretical schematic of the Epistemological Construction Cycle as proposed by the MNIEC. The sequential presentation of the five phases reflects an analytical decomposition for theoretical clarity; actual ECC operation during scientific writing is recursive, overlapping, and temporally nested. The depicted neurobiological correlates represent model-derived predictions, not empirically validated mechanisms.

### 3.4 System failure conditions

A neuropsychological model grounded in clinical reality must specify not only the conditions of optimal functioning but also the conditions under which the system fails. The MNIEC identifies four dysfunction patterns that can compromise the ECC:

1. *Type I Failure***—***Executive saturation*: when low-level demands (spelling, grammar, formatting) saturate the capacity of the CES and EWMS, Phase 2 (working memory simulation) is compromised and the ECC stalls at superficial text production without accessing higher-order conceptual construction. This pattern is characteristic of novice writers and of high time-pressure situations.
2. *Type II Failure—Semantic activation deficit*: an impoverished semantic network or inefficient retrieval from long-term memory may compromise Phase 1, generating what the writer experiences as a “blank mind” or writer's block. The MNIEC predicts that this pattern involves relative DMN hypoactivation with insufficient compensatory activation of the SSS—though it should be noted that writer's block is a heterogeneous phenomenon with multiple potential causes, including motivational depletion, task-switching costs, perfectionism, and insufficient prior domain knowledge, none of which are reducible to a single neurobiological mechanism. The DMN hypothesis constitutes a testable prediction of the model rather than an established explanation.
3. *Type III Failure***—***Metacognitive short-circuit*: in academic impostor syndrome and states of elevated anxiety, the EMMS is predicted to generate error signals of disproportionate intensity relative to the actual inconsistencies detected, blocking Phase 5 (conceptual reorganization) and producing repetitive revision cycles without epistemic progression. The MNIEC proposes that this pattern involves amygdalar hyperactivation suppressing the medial prefrontal activity required for calibrated metacognitive monitoring (Pessoa, 2008)—a mechanistic hypothesis derived from the model's architecture rather than a demonstrated explanation of impostor phenomena. It is important to note that academic impostor syndrome is a complex psychological phenomenon involving institutional pressures, identity factors, supervision dynamics, perfectionism, and publication culture, many of which operate independently of the writing cycle itself. Competing psychological accounts—including cognitive-behavioral formulations, emphasizing maladaptive self-evaluation schemas—should be considered alongside the neurobiological hypothesis proposed here.
4. *Type IV Failure***—***Externalization-metacognition dissociation*: when Phase 3 (externalization) operates fluently but Phase 4 (metacognitive evaluation) is compromised—as occurs under conditions of severe cognitive fatigue or when LLMs are used as substitutes for the writing process—the text produced may be superficially coherent yet epistemologically hollow: it reflects not a genuine process of knowledge construction but a reproduction of linguistic patterns without underlying conceptual reorganization.

### 3.5 Differentiation of the MNIEC from existing models

The field of cognitive psychology of writing has produced three reference models constituting the state of the art prior to the MNIEC. A precise understanding of their scope and limitations is necessary to situate the original contribution of the present model (see Table 1).

**Table 1:** Comparative analysis of the MNIEC and preceding cognitive models of writing across key theoretical dimensions.

Dimension	Hayes and Flower (1980)	Kellogg (2008)	Olive (2014)	MNIEC
Neurobiological basis	Absent	Implicit	Implicit	Explicitly formalized
Large-scale brain network integration	Absent	Absent	Absent	Explicitly formalized
Epistemological metacognition	Absent	Partially developed	Absent	Explicitly formalized
Extended cognition	Absent	Absent	Absent	Explicitly formalized
Affective-motivational modulation	Implicit	Implicit	Absent	Explicitly formalized
System failure conditions	Absent	Partially developed	Absent	Explicitly formalized
Specific to scientific writing	Partially developed	Partially developed	Partially developed	Explicitly formalized
Predictive coding framework	Absent	Absent	Absent	Explicitly formalized

Comparative evaluation based on each model's original formulation. Absent: the dimension is neither present nor implied in the model. Implicit: the dimension is suggested but not developed. Partially developed: the dimension is present but lacks formal specification. Explicitly formalized: the dimension is formally defined, operationalized, and constitutive of the model's architecture. It should be noted that the MNIEC's ratings reflect theoretical proposals rather than empirically validated mechanisms; the table indicates what the model formally specifies, not what has been empirically demonstrated. MNIEC: Integrated Neuropsychological Model of Scientific Writing. Table of own elaboration based on Hayes and Flower (1980), Kellogg (2008), and Olive (2014).

The model of Hayes and Flower (1980) was the first to propose an explicit cognitive architecture of the writing process, articulated around three central processes—planning, translating, and revising—under the control of an executive monitor, demonstrating that writing is not linear but hierarchically organized. However, it lacks neurobiological grounding, does not incorporate working memory as a multicomponent system, and was designed to account for writing in general, not for scientific writing as a specialized system of knowledge construction. Kellogg's (2008) framework advanced the field by introducing the novice-expert distinction in terms of cognitive resource distribution, yet it lacks neurobiological specificity and offers no explanatory mechanism for how writing generates new knowledge. Olive's (2014) parallel-cascading model more faithfully captures the dynamic nature of expert writing but remains fundamentally a model of text production—not of knowledge construction—without integration with contemporary cognitive neuroscience.

The MNIEC differs from these three models along dimensions that are not merely quantitative. Its central contribution is the proposal of an explicit generative mechanism—the ECC—that articulates neurocognitive systems within a recursive process of epistemological construction, anchored in identifiable neurobiological substrates and amenable to empirical verification. The difference is qualitative and operates at three levels:

1. First, the *level of analysis*: preceding models operate at the level of functional cognitive processes; the MNIEC operates simultaneously at both the functional and neurobiological levels, specifying the neural substrates of each system and each phase of the ECC.
2. Second, the *object of modeling*: existing models model text production; the MNIEC models knowledge construction through text. This distinction is not semantic—it implies entirely different mechanisms, particularly in Phase 5 of the ECC (conceptual reorganization) and in the role of the EMMS.
3. Third, the *explanatory scope*: the MNIEC integrates dimensions that preceding models do not address—extended cognition (Clark and Chalmers, 1998), affective-motivational modulation (Pessoa, 2008), large-scale brain network dynamics (Menon and Uddin, 2010), and the predictive coding framework (Friston, 2010)—constituting an explanatory system of broader reach and greater empirical specificity.

This differentiation does not imply that prior models are incorrect—they are valid within their own frames of reference. The MNIEC integrates them as special cases within a broader architecture, in the same way that relativistic mechanics does not invalidate Newtonian mechanics but delimits its conditions of applicability with greater precision.

## 4 Testable hypotheses and experimental proposals

The MNIEC is a theoretical model whose scientific validity depends on its capacity to generate empirically testable predictions. Four hypotheses are derived directly from the model's architecture, each accompanied by a specific experimental paradigm for empirical validation.

### 4.1 Hypothesis 1—Differential brain network activation across ECC phases

#### 4.1.1 Hypothesis

The distinct phases of the ECC produce qualitatively different and dissociable neurobiological activation patterns, detectable through functional neuroimaging. Specifically, Phase 1 (conceptual activation) and Phase 2 (working memory simulation) are predicted to show greater DMN and angular gyrus activation; Phase 3 (symbolic externalization) is predicted to preferentially activate Broca's area, the premotor cortex, and perisylvian language areas, along with enhanced functional connectivity between frontal and temporal language regions as assessed through psychophysiological interaction analyses or dynamic causal modeling—rather than direct BOLD activation of white matter tracts such as the arcuate fasciculus, which would require diffusion tensor imaging or resting-state tractography approaches; and Phase 4 (metacognitive evaluation) is predicted to produce robust activations in the mPFC, rACC, and anterior prefrontal cortex (BA10).

#### 4.1.2 Experimental paradigm

A block-design fMRI study in which participants with high scientific writing expertise (*N* ≥ 30, researchers with verifiable publication records) complete a scientific writing task structured into discrete, externally cued phases (idea generation → simulation → drafting → revision). Phase boundaries will be demarcated by visual cues. A critical methodological challenge concerns the validity of this phase-cueing procedure: given that the ECC is theoretically recursive and overlapping rather than strictly sequential, externally imposed phase boundaries may capture task-induced states rather than naturally occurring ECC phases. To address this, the paradigm will incorporate concurrent process measures—including keystroke logging to track writing fluency and revision patterns, and think-aloud protocols in a separate behavioral session—to verify that the externally cued phases correspond to the qualitatively distinct cognitive processes they are intended to operationalize. The text produced at each phase will be subsequently analyzed for linguistic and semantic markers consistent with the predicted cognitive mode. Phase-by-phase activation contrasts will be computed using fixed-effects GLM and random-effects group analysis. Control variables: writing speed, text length produced, and expertise level (assessed via h-index and years of research experience).

### 4.2 Hypothesis 2—Cognitive amplification through symbolic externalization

#### 4.2.1 Hypothesis

The availability of written externalization during scientific writing amplifies the conceptual complexity of the knowledge produced, beyond what is predicted by individual internal working memory capacity. Specifically, the argumentative complexity of text produced under free-writing conditions is predicted to significantly exceed that produced under blocked-writing conditions (in which the written text is not available for re-reading). Critically, this externalization advantage is predicted to interact with individual working memory capacity: writers with lower WM capacity are expected to show a larger benefit from externalization than writers with higher WM capacity, consistent with the MNIEC's principle of symbolic amplification—the written text functions as a cognitive extension that compensates for the limits of internal WM. This interaction prediction is theoretically central: if the externalization advantage were uniform across WM capacity levels, it would suggest that writing functions merely as a transcription medium rather than as a genuine amplifier of cognitive capacity.

#### 4.2.2 Experimental paradigm

A mixed-design behavioral study (*N* ≥ 80 graduate students). Condition 1: free writing with continuous access to the text produced. Condition 2: blocked-screen writing (the text disappears after each sentence). Condition 3: oral dictation without visual feedback. Dependent variable: argumentative complexity of the text, assessed through global coherence analysis (CohMetrix software; CohMetrix, University of Memphis, Memphis, TN, United States; McNamara et al., 2014), propositional density, and number of explicit causal relations. Blocking independent variable: reading span (RSPAN) and WM updating (2-back task). A significant Condition × WM capacity interaction is predicted: the difference between conditions will be smaller for high-WM writers than for low-WM writers, confirming the amplifying effect of externalization particularly when internal resources are limited. This interaction prediction is the primary test of the symbolic amplification principle; a null interaction—in which the externalization advantage is equivalent across WM capacity levels—would constitute evidence against this specific claim of the MNIEC and would require revision of Principle 3. Future studies should include power analyses based on medium-to-large interaction effect sizes (*f*^2^ ≥ 0.15), given the theoretical importance of detecting or ruling out this effect.

### 4.3 Hypothesis 3—Metacognitive efficiency as a predictor of epistemic quality

#### 4.3.1 Hypothesis

Individual metacognitive efficiency—defined as the capacity to discriminate between accurate and inaccurate representations during the revision of one's own text—will significantly predict the epistemological quality of the scientific text produced, beyond measures of general intelligence, verbal fluency, and declarative domain knowledge.

#### 4.3.2 Experimental paradigm

A predictive correlational study (*N* ≥ 100 active researchers). Measures: (a) metacognitive efficiency: a post-decision confidence judgment task adapted to a scientific domain (meta-*d*′/*d*′ ratio; Fleming and Dolan, 2012), administered prior to the writing task to obtain an independent measure of metacognitive capacity unconfounded by writing performance; (b) epistemological text quality: blind expert-peer evaluation using a rubric assessing argumentative coherence, conceptual novelty, and epistemic precision. To ensure measurement rigor, the following procedural standards will be implemented: (i) a minimum of three independent expert raters per text, drawn from researchers active in the participant's disciplinary domain with verifiable publication records; (ii) raters will be blind to participant identity, metacognitive scores, and all other study measures; (iii) inter-rater reliability will be assessed using intraclass correlation coefficients (ICC), with a minimum acceptable threshold of ICC ≥ 0.70 prior to averaging ratings; (iv) the rubric will undergo pilot validation with a separate sample of texts prior to the main study, with item-level reliability and construct validity assessed; (c) covariates: RSPAN score, scientific vocabulary (WAIS-IV Vocabulary subtest), years of research experience, and h-index. Hierarchical block regression will be applied, entering covariates in Block 1 and metacognitive efficiency in Block 2. Metacognitive efficiency is predicted to account for significant incremental variance (Δ*R*^2^ ≥ 0.10) beyond Block 1 covariates—a criterion selected on the basis of conventions in the individual differences literature, where Δ*R*^2^ values in the range of 0.08–0.15 are considered theoretically meaningful for cognitive predictors of complex performance outcomes (see Ackerman and Heggestad, 1997). A pre-registered power analysis targeting 80% power to detect Δ*R*^2^ = 0.10 with α = 0.05 and four covariates in Block 1 yields a required sample size of approximately *N* = 95, consistent with the proposed minimum of *N* ≥ 100.

### 4.4 Hypothesis 4—Dissociation between fluent text production and knowledge construction in LLM-assisted writing

#### 4.4.1 Hypothesis

The use of large language models (LLMs) as substitutes for the scientific writing process—as opposed to their use as targeted support tools—will selectively compromise Phase 5 of the ECC (conceptual reorganization), producing superficially coherent texts without evidence of modification of the author's conceptual representations in long-term memory.

#### 4.4.2 Experimental paradigm

A randomized experimental study (*N* ≥ 60 doctoral students, with sample size determined by power analysis targeting 80% power to detect a medium effect size, *f* = 0.25, across three conditions). Condition A: fully autonomous scientific writing on an assigned topic. Condition B: complete text generation by an LLM with subsequent editing by the participant. Condition C: LLM use restricted to structural suggestions, with autonomous drafting of content.

To control for alternative explanations of condition differences in knowledge transfer, the following procedural controls will be implemented: (I) topic assignment will be randomized and counterbalanced across conditions to control for differential prior knowledge; (II) a domain knowledge pretest will be administered prior to the writing task and included as a covariate in all analyses; (III) total time on task will be equated across conditions using a fixed writing window, with time-on-task recorded via keystroke logging to verify compliance; (IV) in Condition B, the LLM interaction will be standardized through a structured prompting protocol, and the number and depth of editorial interventions will be recorded to quantify actual researcher engagement with the generated text; (V) an immediate transfer test will be administered directly after writing, in addition to the 48-h delayed transfer test, to distinguish encoding effects from retention effects; (VI) participants' subjective sense of ownership and cognitive engagement with the text will be assessed via a brief post-task questionnaire, providing a behavioral correlate of EMMS engagement.

Primary dependent variable: knowledge gain measured through a conceptual transfer test (application of the written content to new problems not covered in the text), with both immediate and delayed (48-h) administrations. Secondary dependent variable: text quality assessed by blinded expert raters using the same rubric as Hypothesis 3. A significant dissociation across conditions is predicted: Condition B is expected to produce texts of surface quality comparable to Condition A, yet will show significantly lower knowledge gain on the delayed transfer test—a pattern consistent with Type IV Failure of the MNIEC. This dissociation would be specifically attributable to the bypassing of Phases 4 and 5 of the ECC rather than to confounds such as time on task or prior knowledge, which the controls described above are designed to rule out.

The four hypotheses derived from the MNIEC are falsifiable in the Popperian sense: they are not *ad hoc* predictions but logically necessary consequences of the model's architecture, whose empirical refutation would require a substantial revision of its nuclear principles. The proposed paradigms constitute a minimal and prioritized research program, designed to test the most central and differentiating mechanisms of the MNIEC relative to existing models. Future research may extend this program to specific populations and to emerging writing modalities that combine human agency with algorithmic assistance. The empirical validation of the MNIEC is not the closure of the model—it is its condition of openness toward a neuropsychology of scientific writing with solid foundations and verifiable applications.

## 5 Discussion

### 5.1 The MNIEC in the context of contemporary cognitive neuroscience

The MNIEC is consistent with the accumulated evidence on the functional organization of the brain into large-scale systems. The dynamic interaction among the frontoparietal control network (FPCN), the default mode network (DMN), and the salience network (SN)—which the ECC formalizes as a recursive cycle of activation, simulation, externalization, evaluation, and reorganization—has been documented in studies of functional connectivity both at rest and during task performance (Menon and Uddin, 2010; Smallwood et al., 2012). The specific contribution of the MNIEC is a functional hypothesis regarding the role of this interaction in scientific knowledge construction: the DMN does not operate as a generic semantic retrieval system but as the neural substrate of the active conceptual simulation that feeds the ECC—an original specification verifiable with currently available neuroimaging paradigms.

The integration of Friston's (2010) predictive coding framework adds further theoretical power. Under the free energy principle, the brain continuously minimizes prediction error between its internal models and available evidence. Applied to the ECC, this principle reframes scientific writing as the iterative minimization of epistemological prediction error across multiple representational levels: propositional coherence, discourse-level argumentative structure, and epistemic validity of the conceptual framework. Each cycle reduces the discrepancy between the writer's internal conceptual model and the externalized text, generating a progressive updating of epistemic beliefs. Empirically, prediction errors at the propositional level might manifest as pausing and revision behavior detectable through keystroke logging; errors at the discourse level might manifest as structural reorganization of sections or arguments; and errors at the epistemic level might manifest as fundamental reconceptualization of the theoretical framework being developed—the highest-order knowledge-constructive effect predicted by the MNIEC. This interpretation is consistent with evidence regarding the role of the anterior prefrontal cortex in generating high-level abstract predictions (Koechlin and Summerfield, 2007), and opens an entirely new line of inquiry at the intersection of computational neuroscience and the psychology of writing. From a learning science perspective, this reframing positions the ECC as a metacognitive self-regulation mechanism operating at the intersection of knowledge construction and epistemic monitoring—a contribution directly relevant to research on metacognition in higher-order learning contexts.

### 5.2 Scientific writing and knowledge generation: implications for epistemic cognition

One of the most significant contributions of the MNIEC is the provision of an explicit neuropsychological mechanism for a phenomenon that had been empirically observed but not satisfactorily explained: that scientific writing, under conditions of sustained metacognitive engagement, can generate knowledge that would not have been accessible without the externalization and recursive evaluation of thought in written form. Klein (1999), in a seminal review, identified multiple pathways through which writing facilitates learning and knowledge construction, yet acknowledged that the underlying cognitive mechanisms remained insufficiently specified. The ECC provides precisely that specification: new knowledge emerges in Phase 5 (conceptual reorganization) as a consequence of the metacognitive evaluation of the externalized text—a process that cannot occur without prior externalization, and that produces a genuine modification of representations in long-term memory.

This mechanism carries direct implications for understanding epistemic cognition in scientific contexts. If scientific knowledge emerges partly from the writing process itself—not solely from prior observation or reasoning—then the quality of the writing process, its executive depth, semantic richness, and metacognitive rigor, constitutes a determining variable in the quality of the knowledge produced. This conclusion converges with the central argument of the *Nature Reviews Bioengineering* editorial (Editorial, 2025), which contends that scientific writing is irreplaceable precisely because it constitutes the cognitive process through which researchers think—not the medium through which they communicate what has already been thought. The MNIEC provides the neuropsychological architecture that grounds this claim beyond philosophical intuition.

This mechanism has direct implications for the understanding of scientific writing as a self-regulated learning process. Within the framework of self-regulated learning (SRL), Zimmerman (2000) conceptualized academic performance as the product of cyclical processes of goal-setting, strategy use, self-monitoring, and self-evaluation. The MNIEC's ECC maps directly onto this SRL cycle: Phases 1 and 2 correspond to forethought and planning; Phase 3 to performance and strategy deployment; and Phases 4 and 5 to self-reflection and adaptive reorganization. Crucially, the MNIEC specifies the neurobiological substrates of each SRL phase in the scientific writing domain—a level of mechanistic grounding that SRL models have not previously achieved. Scientific writing, under this framework, is not merely a product of self-regulated learning: it is one of its most powerful instruments.

From a broader perspective, the MNIEC engages with the tradition of epistemic cognition developed by Kitchener (1983) and subsequently elaborated by Greene et al. (2010), which distinguishes among first-order knowledge (what one knows about the world), metacognitive knowledge (what one knows about one's own knowledge), and epistemic cognition (how one evaluates the justification and limits of one's own beliefs). The EMMS, and in particular the second-order epistemological monitoring the MNIEC attributes to it, corresponds precisely to the level of epistemic cognition in this taxonomy—suggesting that the MNIEC is not only a model of writing but a model of epistemic cognition in action. This alignment suggests that cultivating second-order epistemological monitoring in scientific writers constitutes a high-leverage target for metacognitive training interventions within learning environments.

### 5.3 Affective modulation and scientific cognition: a necessary integration

The explicit incorporation of affective-motivational modulation into the MNIEC addresses a systematic limitation of prior cognitive models of writing, which have operated under the implicit assumption of a rationally neutral writer. The available neurobiological evidence decisively invalidates this assumption.

Pessoa (2008) demonstrated that affective and cognitive systems are deeply integrated at the level of neural circuits. The amygdala does not merely process fear and threat—it actively modulates the availability of prefrontal cortical resources as a function of the emotional valence and intensity of the context. In scientific writing, this implies that evaluation anxiety, fear of editorial rejection, or perception of epistemic incompetence are not contextual variables that merely “interfere” with cognition: they are neurobiological modulators that directly alter the efficiency of the CES, the capacity of the EWMS, and the calibration of the EMMS.

This integration carries first-order clinical and educational consequences. Academic impostor syndrome—characterized by a persistent inability to internalize one's own achievements and by chronic fear of being ‘discovered' as incompetent (Clance and Imes, 1978)—may be partially reframed within the MNIEC as a Type III Failure pattern: the model predicts that the affective dysregulation characteristic of impostor phenomena involves amygdalar hyperactivation that suppresses the medial prefrontal activity required for calibrated metacognitive monitoring, thereby blocking Phase 5 of the ECC and producing repetitive revision cycles without epistemic progression. This neurobiological account is a hypothesis generated by the model's architecture, not an established explanation of impostor phenomena, and it should be understood as complementary to—rather than replacing—psychological accounts that emphasize identity, perfectionism, and institutional dynamics. Identifying this pattern in neuropsychological terms opens the possibility of interventions grounded in emotional regulation and metacognitive training, with direct impact on researcher scientific productivity.

The dopaminergic reward system adds a further dimension. Expert scientific writing generates epistemological predictions that, upon confirmation or disconfirmation through metacognitive evaluation, produce reward prediction error signals that modulate the motivation to continue the cycle (Schultz, 1997). Writers who experience writing as intrinsically rewarding—not for its external consequences (publication, recognition) but for the epistemic pleasure of the conceptual construction process—show greater persistence, higher tolerance for ambiguity, and greater willingness to radically reorganize their representations in Phase 5. This observation is consistent with the literature on intrinsic motivation and scientific creativity (Amabile, 1996), and suggests that the design of scientific training environments should attend not only to the development of executive and metacognitive competencies, but to the cultivation of intrinsic epistemic motivation as a neurobiological condition for optimal ECC performance. This neurobiological account of intrinsic motivation in scientific writing aligns with Efklides' (2011) metacognitive and affective model of self-regulated learning, which positions affective metacognitive experiences—feelings of difficulty, confidence, and satisfaction—as central regulators of learning effort and persistence.

### 5.4 The MNIEC in the face of large language models

No discussion of the neuropsychology of scientific writing can avoid, at this historical moment, the question of the impact of large language models (LLMs) on scientific writing practice. The MNIEC offers a precise conceptual framework for evaluating this impact—beyond normative debates on ethics or academic integrity—in terms of its consequences for researcher cognition.

Hypothesis 4 of the model predicts a specific dissociation: the use of LLMs as substitutes for the writing process will produce superficially coherent texts without evidence of conceptual reorganization in the author's long-term memory. This prediction is consistent with the observation of the *Nature Reviews Bioengineering* editorial (Editorial, 2025) regarding the risk that outsourcing writing to LLMs may deprive researchers of the generative cognitive function that the writing process exercises. A systematic synthesis of recent evidence on generative AI in academic writing confirms that unstructured LLM use is associated with reduced metacognitive engagement and shallower knowledge integration, while reflective or dialogic LLM use can support deeper learning—a pattern directly convergent with the MNIEC's continuum from substitutive to heuristic AI use (Sanz-Tejeda et al., 2026). Survey evidence from knowledge workers further documents self-reported reductions in cognitive effort and critical thinking engagement associated with heavy generative AI reliance—particularly in tasks requiring active knowledge construction—consistent with the model's prediction of Phase 4 and 5 compromise under substitutive LLM use (Lee et al., 2025). Within the MNIEC, this observation acquires a mechanistic specification at the level of prediction: what the model anticipates is lost when writing is outsourced to an LLM is not the motor effort of typing—but Phase 4 (metacognitive evaluation) and Phase 5 (conceptual reorganization) of the ECC, precisely the phases through which writing can function as a mechanism of knowledge construction. This prediction has not yet been empirically verified and constitutes one of the model's most directly testable claims, formalized as Hypothesis 4.

It is important to note, however, that the MNIEC does not predict that any use of LLMs in scientific writing is cognitively detrimental. Rather than constituting fixed and mutually exclusive categories, LLM use in scientific writing is better understood as a continuum ranging from maximal cognitive offloading—in which the researcher delegates epistemic agency to the model and engages only in superficial editing—to dialogic scaffolding—in which the researcher uses the LLM as a reflective interlocutor while maintaining active metacognitive control over the conceptual process. For analytical clarity, three regions of this continuum can be distinguished: (a) *substitutive use*, in which the LLM generates the text and the researcher performs only superficial editing—this mode is predicted to compromise Phases 4 and 5 of the ECC by bypassing the metacognitive evaluation and conceptual reorganization that constitute the model's knowledge-constructive mechanism; (b) *instrumental use*, in which the LLM assists with low-level tasks (grammatical correction, reference reformatting, readability improvement) while the researcher retains control over the conceptual process—this mode is predicted to be neuropsychologically neutral or positive, as it may free executive and working memory resources for the phases of greatest cognitive demand; and (c) *heuristic use*, in which the LLM generates conceptual alternatives or identifies unforeseen connections that the researcher evaluates critically—this mode is predicted to potentially enrich Phase 1 of the ECC (conceptual activation) and, if the researcher maintains active EMMS engagement during evaluation, may support rather than bypass the knowledge-constructive function of the ECC. In practice, individual writing sessions may traverse multiple regions of this continuum, and the cognitive consequences of LLM use are likely to vary as a function of the researcher's expertise level, metacognitive awareness, and the specific phase of the ECC in which the tool is engaged.

This taxonomy carries direct implications for the design of editorial and institutional policies on the use of AI in scientific research—policies that, at the time of writing, remain under definition in the majority of academic institutions worldwide. The MNIEC predicts that the relevant criterion is not whether an LLM was used, but whether the writing process preserved the integrity of the ECC—that is, whether the researcher maintained executive, metacognitive, and epistemological control over the knowledge construction process. This prediction awaits empirical verification, but it offers a neuropsychologically grounded criterion for the design of institutional and editorial policies on AI-assisted scientific writing.

### 5.5 Limitations of the model and future directions

The MNIEC is a first-generation theoretical model and presents limitations that must be explicitly acknowledged.

First, the neurobiological specification of the model rests on the extrapolation of findings from studies on general writing, executive functions, working memory, and metacognition—not from studies specifically designed to investigate scientific writing as an integrated neurocognitive system. Scientific writing imposes specific demands—logico-formal argumentation, management of epistemic uncertainty, citation practices, and disciplinary positioning—that may recruit partially different neural substrates from those identified in general writing paradigms. The experimental paradigms proposed in Section 4 are designed precisely to generate the specific neurobiological evidence that would allow this specification to be refined.

Second, the model does not specify the temporal parameters of the ECC—the duration of each phase, the iteration frequency, and their variation as a function of expertise level. Such specification would require studies combining high temporal resolution measures (EEG, MEG) with keystroke logging—a methodology that has proven particularly productive in writing process research (Leijten and Van Waes, 2013).

Third, the model does not systematically address the sociocultural dimension of scientific writing—the rhetorical conventions of the disciplinary field, the expectations of the epistemic community, and the power asymmetries that structure academic communication (Hyland, 2004)—nor its applicability to researchers writing in a non-native language or in collaborative writing contexts, where the ECC is distributed across multiple cognitive agents. From an applied perspective, the MNIEC's implications for writing-task design in scientific training remain to be operationalized. Meta-analytic evidence demonstrates that writing activities facilitate content learning, but that the magnitude of this effect depends critically on the cognitive demands imposed by the task: writing assignments that require explanation, conceptual transformation, active revision, and learner responsibility for content generation produce substantially larger learning gains than tasks that permit reproduction or reformatting of stabilized knowledge (Bangert-Drowns et al., 2004; Graham et al., 2020). Applied to the MNIEC's architecture, this finding suggests that ECC-informed task design in scientific training programs should prioritize assignments that impose genuine epistemic demands—specifically, tasks that engage Phases 4 and 5 of the ECC (metacognitive evaluation and conceptual reorganization) rather than tasks that can be completed through surface-level text manipulation. A neuropsychologically grounded taxonomy of writing-task demands, derived from the MNIEC's system architecture, constitutes a promising direction for future research at the intersection of cognitive neuroscience and scientific writing pedagogy.

A fourth limitation concerns the boundary conditions of the model. The MNIEC was developed with primary reference to high-level scientific writing—specifically, the kinds of writing that require sustained argumentative construction, theoretical integration, conceptual synthesis, and recursive epistemic evaluation: theory-building manuscripts, integrative literature reviews, and original empirical papers in which the framing and interpretation of findings involve substantive conceptual work. The model's claims are strongest for these genres. Its applicability to other forms of scientific writing—including methods sections, structured empirical reports with constrained formats, systematic reviews following predefined protocols, grant applications, preregistrations, and collaborative manuscripts in which the ECC is distributed across multiple cognitive agents—requires separate examination. These genres differ substantially in the degree to which they engage Phases 4 and 5 of the ECC (metacognitive evaluation and conceptual reorganization), which are the phases through which the model predicts the strongest knowledge-constructive effects. Similarly, the model does not yet address the additional cognitive and affective demands of scientific writing in a non-native language, a context in which low-level linguistic processing may compete more intensively for the executive and working memory resources required for higher-order conceptual construction.

These limitations do not undermine the validity of the MNIEC as a theoretical proposal—they constitute the conditions of its future scientific productivity. A model that explained everything while leaving no open questions would not be a scientific model: it would be a description.

## 6 Conclusions

The present paper has proposed the Integrated Neuropsychological Model of Scientific Writing (MNIEC, from its Spanish acronym) as a theoretical framework that reconceptualizes scientific writing—from terminal communicative act to active neurocognitive system of knowledge construction. This reconceptualization implies identifiable neurobiological mechanisms, empirically verifiable predictions, and practical consequences of significant reach for scientific training, editorial policy, and the evaluation of the cognitive impact of automated text-generation technologies.

The MNIEC integrates four neurocognitive systems—the CES, the EWMS, the SSS, and the EMMS—within a unified architecture whose central operation is the Epistemological Construction Cycle (ECC): a recursive five-phase process—conceptual activation, working memory simulation, symbolic externalization, metacognitive evaluation, and conceptual reorganization—through which scientific writing can produce new knowledge—particularly when recursive metacognitive evaluation and conceptual reorganization are actively engaged rather than bypassed. Five nuclear principles guide this architecture—epistemic emergence, constructive recursivity, symbolic amplification, affective-motivational modulation, and expertise sensitivity—and constitute conditions of internal coherence of the model, not auxiliary assumptions. Among them, the principle of symbolic amplification neuropsychologically formalizes the extended mind thesis (Clark and Chalmers, 1998): the written text is a constitutive part of the researcher's cognitive system during writing, and its availability qualitatively amplifies the complexity of the knowledge the system is capable of constructing.

The integration of the predictive coding framework (Friston, 2010) reframes the ECC as a process of iterative minimization of epistemological prediction error—an interpretation that connects the MNIEC with the most influential theoretical current in contemporary computational neuroscience and generates specific predictions verifiable through functional neuroimaging.

The MNIEC differs from preceding models—Hayes and Flower (1980), Kellogg (2008), Olive (2014)—through a qualitative shift in the level of analysis and in the object of modeling: whereas prior models describe text production, the MNIEC models knowledge construction through text, operating simultaneously at the functional and neurobiological levels. This distinction situates earlier models as special cases within a broader explanatory architecture, not as incorrect formulations.

The affective-motivational modulation incorporated into the model carries immediate applied consequences: academic impostor syndrome, evaluation anxiety, and cognitive fatigue can be reframed as ECC failure patterns with identifiable neurobiological bases, addressable through emotional regulation and metacognitive training interventions. In the context of large language models, the MNIEC offers a precise neuropsychological criterion: the relevant question is not whether algorithmic assistance was used in the writing process, but whether the writing process preserved the integrity of Phases 4 and 5 of the ECC—those responsible for conceptual reorganization and the generation of new knowledge.

The MNIEC is a first-generation model whose limitations—the absence of temporal specification of the ECC, the insufficient integration of the sociocultural dimension, and the need for direct empirical validation—constitute the map of a research agenda, not weaknesses to be concealed. A theoretical model is valued by the new questions it generates. The MNIEC generates questions that have not yet been systematically addressed within the cognitive neuroscience of writing.

Empirical validation of the MNIEC's predictions will determine the extent to which this reconceptualization holds across writing genres, expertise levels, and cultural contexts. Scientific writing is not a passive mirror of knowledge—it is an active instrument of its construction. Understanding the neuropsychological mechanisms through which this occurs is a condition for preserving, in an increasingly automated world, the irreplaceable specificity of human scientific cognition.
